# Pressure coefficients for direct optical transitions in MoS_2_, MoSe_2_, WS_2_, and WSe_2_ crystals and semiconductor to metal transitions

**DOI:** 10.1038/srep26663

**Published:** 2016-05-24

**Authors:** F. Dybała, M. P. Polak, J. Kopaczek, P. Scharoch, K. Wu, S. Tongay, R. Kudrawiec

**Affiliations:** 1 Faculty of Fundamental Problems of Technology, Wroclaw University of Technology, Wybrzeże Wyspiańskiego 27, 50-370 Wrocław, Poland; 2 School for Engineering of Matter, Transport and Energy, Arizona State University, Tempe, Arizona 85287, United States

## Abstract

The electronic band structure of MoS_2_, MoSe_2_, WS_2_, and WSe_2_, crystals has been studied at various hydrostatic pressures experimentally by photoreflectance (PR) spectroscopy and theoretically within the density functional theory (DFT). In the PR spectra direct optical transitions (A and B) have been clearly observed and pressure coefficients have been determined for these transitions to be: α_A_ = 2.0 ± 0.1 and α_B_ = 3.6 ± 0.1 meV/kbar for MoS_2_, α_A_ = 2.3 ± 0.1 and α_B_ = 4.0 ± 0.1 meV/kbar for MoSe_2_, α_A_ = 2.6 ± 0.1 and α_B_ = 4.1 ± 0.1 meV/kbar for WS_2_, α_A_ = 3.4 ± 0.1 and α_B_ = 5.0 ± 0.5 meV/kbar for WSe_2_. It has been found that these coefficients are in an excellent agreement with theoretical predictions. In addition, a comparative study of different computational DFT approaches has been performed and analyzed. For indirect gap the pressure coefficient have been determined theoretically to be −7.9, −5.51, −6.11, and −3.79, meV/kbar for MoS_2_, MoSe_2_, WS_2_, and WSe_2_, respectively. The negative values of this coefficients imply a narrowing of the fundamental band gap with the increase in hydrostatic pressure and a semiconductor to metal transition for MoS_2_, MoSe_2_, WS_2_, and WSe_2_, crystals at around 140, 180, 190, and 240 kbar, respectively.

Recently a few-layer transition metal dichalcogenides (TMDs), such as MoS_2_, MoSe_2_, WS_2_, and WSe_2_, have garnered significant interest based on their unique mechanical and optical properties^1^,^2^,^3^,^4^,^5^. MoS_2_, MoSe_2_, WS_2_, and WSe_2_ are layered crystals in a hexagonal structure consisting of sixfold-bonded metal atoms sandwiched between two threefold-bonded chalcogenide atoms with a covalent interaction between atoms within a layer and a van der Walls interaction between layers. In general, bulk TMDs have been investigated in the past^6^,^7^, but since the very first paper on MoS_2_ layers^1^, it is well established that the electronic band structure of TMDs strongly varies with the number of layers and exhibits indirect-to-direct band gap transition with the size reduction to a single layer. In recent years a lot of works has been devoted to theoretical studies of the electronic band structure of TMDs of various number of layers^8^,^9^. Moreover numerous theoretical studies have discussed the influence of hydrostatic pressure and strain on band structure of few-layers TMDs and notable physical effects such as strain-induced direct-to-indirect band gap transition and semiconductor to metal transition have been predicted^10^,^11^,^12^,^13^,^14^,^15^,^16^,^17^,^18^,^19^,^20^,^21^,^22^. Unfortunately experimental studies of these phenomena are very limited^23^,^24^,^25^ since measurements of a single layer of TMDs are still a challenge, especially at high hydrostatic pressures. Moreover it is worth noting that even bulk TMDs have not been experimentally at high hydrostatic pressures and therefore pressure coefficients for indirect gap and direct optical transitions in MoS_2_, MoSe_2_, WS_2_, and WSe_2_ are rather unknown. Recently a semiconductor to metal transition in multilayer MoS_2_ has been observed by applying hydrostatic pressure^26^. Very similar phenomenon was reported for multilayered WS_2_^27^. It means that pressure induced changes in the electronic band structure of bulk TMDs are a very interesting issue to explore. They were not intensively investigated up to now and therefore they are the aim of our studies.

In this paper to investigate the electric band structure of MoS_2_, MoSe_2_, WS_2_, and WSe_2_ under hydrostatic pressure we applied photoreflectance (PR) spectroscopy. This technique due to its absorption-like character is an excellent tool to investigate the direct optical transitions between both ground and excited states^28^,^29^,^30^. The differential-like character of PR spectroscopy allows to eliminate the background signal and detect even very weak optical transitions. So far PR spectroscopy has been successfully applied to study the optical transitions in bulk MoS_2_^31^, but this technique has never been applied to study optical transitions in such TMDs as MoSe_2_, WS_2_, and WSe_2_. In this work PR spectroscopy has been applied to determine the pressure coefficients for direct optical transitions in MoS_2_, MoSe_2_, WS_2_, and WSe_2_ in the range of hydrostatic pressure <20 kbar. Experimental results are compared with those calculated from first principles within the density functional theory (DFT), where different approaches to the calculations of TMDs are carefully studied and compared. In addition, the hydrostatic pressure for which the semiconductor to metal transition is expected in MoS_2_, MoSe_2_, WS_2_, and WSe_2_ is estimated.

## Results and Discussion

**Figure 1:**
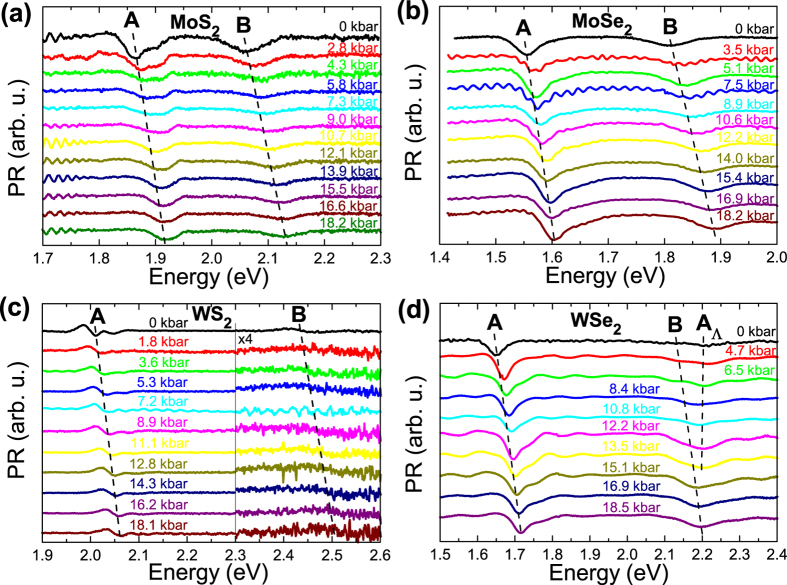
Room temperature PR spectra measured for MoS_2_ (**a**), MoSe_2_ (**b**), WS_2_ (**c**), and WSe_2_ (**d**) crystals at various hydrostatic pressures. The A transition is very clearly visible for all four crystals. The B transition is very clearly visible for sample MoS_2_ and MoSe_2_. For sample WS_2_ the B transition is weaker in comparison to the A transition and therefore the high energy part of PR spectra is multiplied by a factor of 4. For sample WSe_2_ an extra transition labeled as A_Λ_ is visible near the B transition. The A_Λ_ transition is stronger than B at low hydrostatic pressures but with the increase in the hydrostatic pressure its intensity decreases and its spectral position does not shifts significantly. Dashed lines schematically illustrates shift of labeled optical transitions with the increase in the hydrostatic pressure.

**Figure 2:**
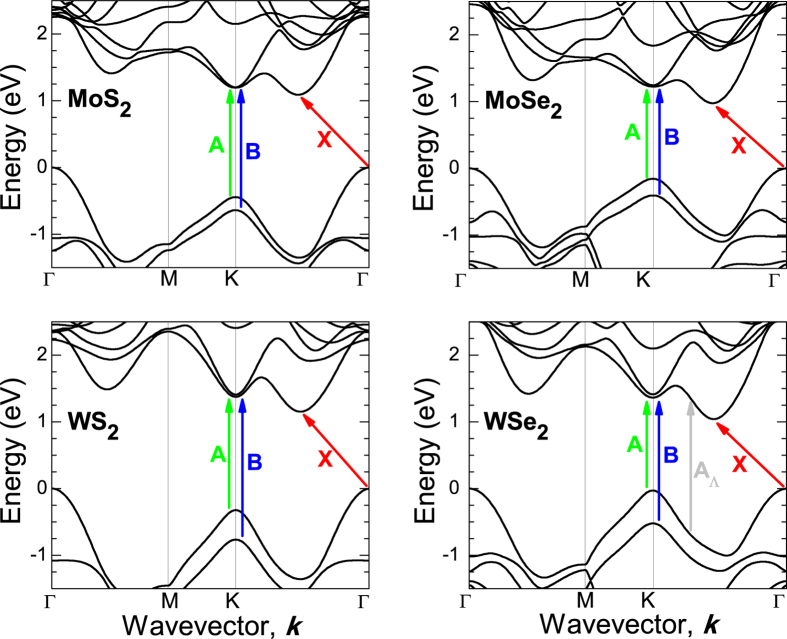
Electronic band structure for MoS_2_ (**a**), MoSe_2_ (**b**), WS_2_ (**c**), and WSe_2_ (**d**) crystals with marked optical transitions A, B, and X, calculated with MBJLDA functional and GGA + DFT-D3 geometry optimization.

Figure 1(a–d) show PR spectra measured at various hydrostatic pressures for MoS_2_, MoSe_2_, WS_2_, and WSe_2_ samples, respectively. For each sample two optical transitions (labeled as A and B in Fig. 1) are clearly visible in the PR spectra. The origin of these transitions is identified in Fig. 2 where the electronic band structure is plotted for the four samples. According to Fig. 2, transitions A and B are direct optical transitions at K point of Brillouin zone. The stronger intensity of the A transition in comparison to the B transition means a stronger singularity in the optical joint density of states for the A transition. Very similar situation is observed for III-V semiconductors (e.g. GaAs or InAs) where the intensity of the fundamental transition between the heavy/light hole bands and the conduction band is stronger that the intensity of the optical transition between the spin-orbit split-off band and the conduction band^29^,^30^.

With the increase in the hydrostatic pressure, both A and B transition shift to higher energies due to pressure induced changes in the electronic band structure of TMDs. The weak oscillation in some PR spectra (see for example blue curve in Fig. 1(a)) is associated with the Fabry-Perot oscillation on a liquid film which appears between the sample and the window in the pressure cell. This spectral feature is unwanted but fortunately does not influence the analysis of PR spectra. In order to determine the pressure coefficients for direct optical transitions in studied samples, energies of A and B transitions have to be extracted from PR spectra. It can be done by fitting PR spectra by Aspnes’ formula^32^ and/or using Kramers-Kroning analysis (KKA)^33^.

Aspnes’s formula is widely applied to analyze PR spectra^28^,^29^,^30^,^31^ and is given by Eq. (1)


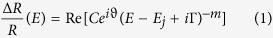


where 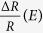 is the energy dependence of PR signal, C and ϑ are the amplitude and phase of the line, and *E*_*j*_ and Γ are the energy and the broadening parameter of the optical transition, respectively. The term *m* for excitonic transition equals 2.

**Figure 3:**
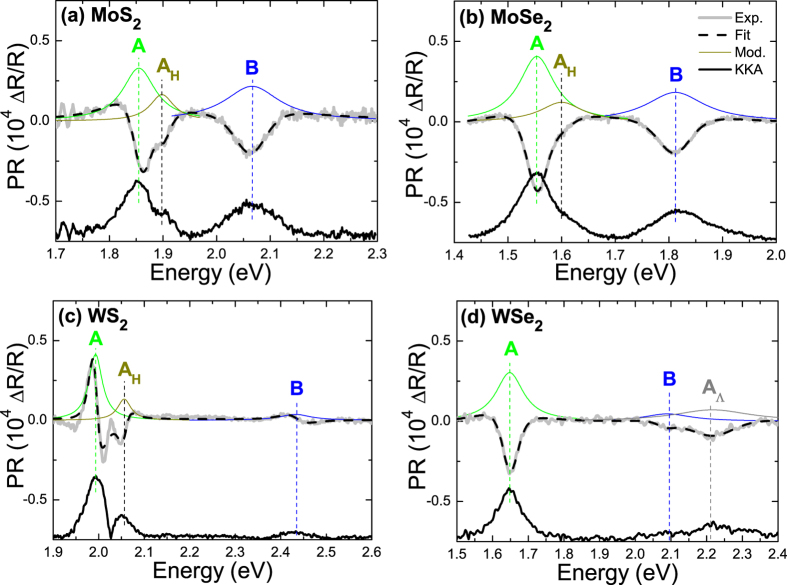
PR spectra measured at atmospheric pressure together with fitting curves (dashed lines) and modulus of individual PR resonances (solid lines).Thick solid black lines correspond to KKA of PR spectra.

Figure 3 shows the PR spectra measured at atmospheric pressure for MoS_2_, MoSe_2_, WS_2_, and WSe_2_ sample together with the fitting curves (dashed lines) and the moduli of individual PR resonances. The moduli of PR resonance have been obtained according to Eq. (2) with parameters taken from the fit.


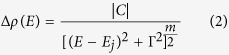


**Figure 4:**
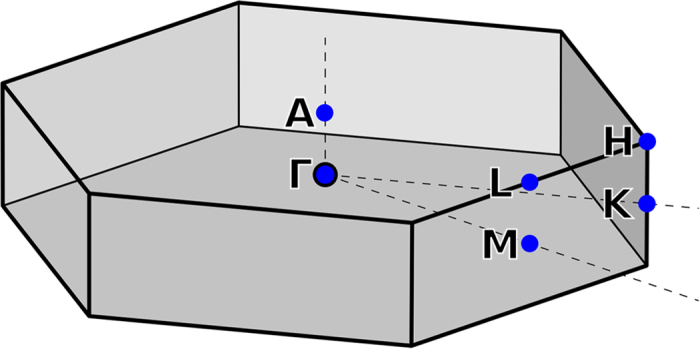
Brillouin zone of 2H-MX_2_ (M = Mo or W and X = S or Se).

For samples MoS_2_, MoSe_2_, and WS_2_ an extra PR resonance is visible at high energy side of transition A (see the optical transition labeled as A_H_). It is worth noting that similar spectral features have been observed for MoS_2_ and WS_2_ in piezoreflectance spectra^34^,^35^,^36^ where the authors have attributed this signal to an excited state of A exciton transition. However recent PR studies of MoS_2_ crystals suggest that this resonance is associated with a direct optical transition at H point of Brillouin zone^31^, see the Brillouin zone in Fig. 4. Our DFT calculations of the electronic band structure for TMDs confirm this interpretation and therefore A_H_ transition is attributed to the direct optical transition at H point of Brillouin zone^31^. A direct optical transition at H point is also expected near transition B (a B_H_ transition), but the energy separation between B and B_H_ transition is small (it is <30 meV according to our DFT calculations) and therefore B and B_H_ transitions are not resolved in PR spectra. The same situation is for A and A_H_ transition in WSe_2_ sample. These two transitions are not resolved due to small energy separation between them and a significant broadening for these transitions. However for WSe_2_ sample an extra resonance has been added to simulate PR spectrum in the region of B transition. This resonance is not associated with B_H_ transition since its spectral position is too high (this transition is observed ~120 meV above B transition). According to our calculations of the electronic band structure for this crystal it could be an optical transition near K point of the Brillouin zone, see transition labeled as A_Λ_ in Fig. 3(d). According to our calculations A_Λ_ transition is expected ~120 meV above B transition that is very consistent with the analysis of PR spectrum for this sample. It is worth noting that similar band nestings are also present for remaining TMDs but optical transitions at these points are expected at higher energies than B transition and therefore they do not interfere with B transition in PR spectra. The A_Λ_ transition for WSe_2_ sample is not the subject of this paper and therefore its pressure dependence is not discussed in this work.

The character of A, A_H_, and B transitions observed in PR spectra is excitonic since the exciton binding energy in these crystals is comparable with the thermal energy at room temperature or even higher^37^,^38^,^39^. To study the pressure coefficients we focus on A and B transitions. Energies of these transitions can be also determined using KKA. In this approach the complex PR function is defined as


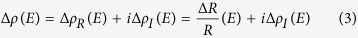


where the imaginary part of this function can be calculated from the Kramers-Kronig relations by


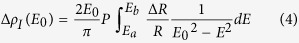


where the symbol *P*∫ means the principal value of the integral. *E*_*a*_ and *E*_*b*_ determine the energy range in which Δ*R/R* is measured. The values of these energy limits occur when Δ*R/R*(*E*_*a*_) and Δ*R/R*(*E*_*b*_) are both equal to zero. Knowing the imaginary part of the complex PR spectrum allows determination of the modulus of the PR spectrum according to the following equation


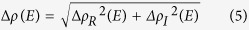


Energies of PR transitions correspond to peaks observed in the modulus (Δ*ρ*) of PR spectrum. The advantage of such analysis is that it bypasses the fitting procedure, which makes the knowledge of the PR line shape unnecessary^40^. KKA of PR spectra measured at atmospheric pressure are shown by thick black lines in Fig. 3. It is clear that the moduli of PR resonances obtained from Eq. (2) correspond very well with the moduli from Eq. (5).

**Figure 5:**
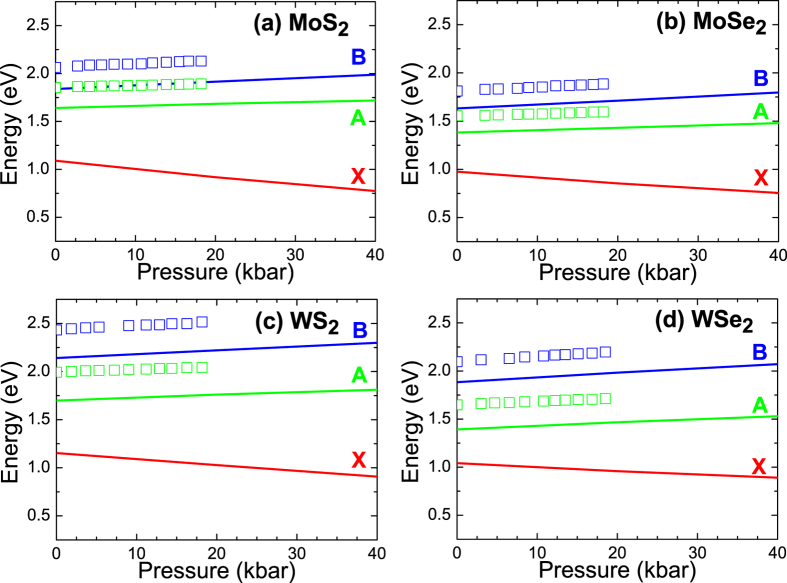
Pressure dependences of energies of A, B and X transitions in MoS_2_ (**a**), MoSe_2_ (**b**), WS_2_ (**c**), and WSe_2_ (**d**) crystals (solid lines) together with experimental data (open points). The lines represent DFT calculations with MBJLDA functional and GGA + DFT-D3 geometry optimization.

In our case both fitting by Aspnes’s formula and the KKA have been applied to determine energies of A and B transitions in the whole range of hydrostatic pressures for the four samples. Energies of A and B transitions extracted from PR spectra are plotted in Fig. 5 (open squares) together with theoretical predictions (solid lines). The qualitative agreement between experimental data and theoretical prediction is very good (i.e., energies of A and B transitions increase with the increase in hydrostatic pressure with nearly identical slopes) but quantitatively the agreement is not ideal. However it is well known that the accuracy of determination of the value of the energy gap in DFT calculations depends on the applied functional^41^,^42^ and the choice of other parameters, which is described and studied in detail further in this text. It means that the absolute values of energies of direct optical transitions in TMDs can be underestimated but their relative changes which are induced by the hydrostatic pressure should give reliable values since the errors associated with the exciton binding energy, different temperature for experimental data and theoretical predictions, and inaccuracies in DFT method can be effectively minimized. It is also worth noticing, that the underestimation is consistent between different materials, and the energy separation between A and B transitions is described correctly, which greatly strengthens the results. Pressure coefficients describe only the relative changes in the electronic band structure and therefore it is very reasonable to analyze these relative changes in energies of A and B transitions.

**Figure 6:**
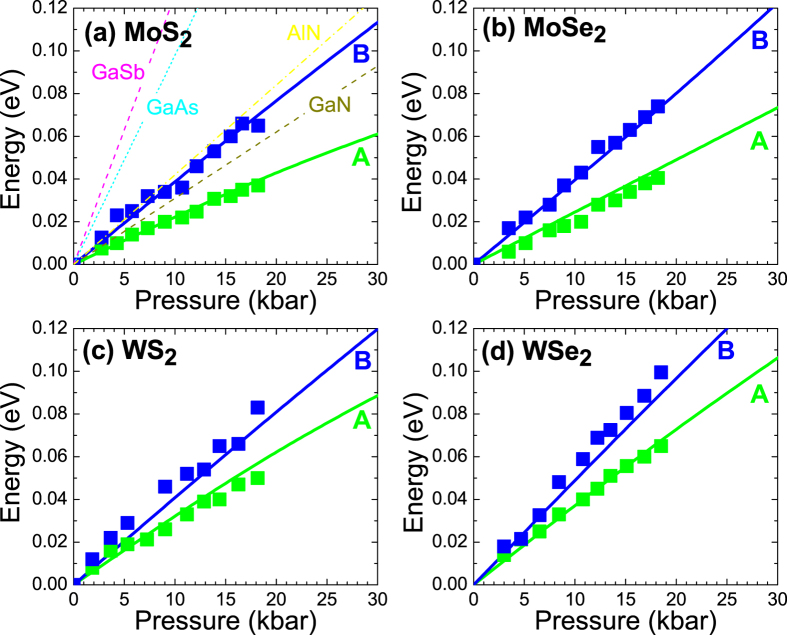
Relative changes in energies of A and B transitions in MoS_2_ (**a**), MoSe_2_ (**b**), WS_2_ (**c**), and WSe_2_ (**d**) crystals (solid lines) together with experimental data (open points). The lines represent DFT calculations with MBJLDA functional and GGA + DFT-D3 geometry optimization.

Figure 6(a–d), show the relative changes in energies of A and B transition for MoS_2_, MoSe_2_, WS_2_, and WSe_2_ sample respectively, together with theoretical predictions for these crystals. It is clearly visible that the agreement between experimental data and theoretical predictions is excellent. This agreement is observed despite the fact that our experimental data correspond to excitonic transitions while theoretical predictions do not take the excitonic effects into account. This is justified since the exciton biding energy does not notably change with hydrostatic pressure. Such assumption is very reasonable since parameters, which determine the exciton binding energy (the dielectric constant and effective masses), do not vary significantly with the increase in the hydrostatic pressure, moreover up to now nobody reported a significant dependence of the exciton binding energy on hydrostatic pressure in any material system. Therefore the direct comparison of experimental data with theoretical predictions, which corresponds to band-to-band transition, is fully acceptable at the first approximation.

The found pressure coefficients are about two times smaller than pressure coefficients for direct band gap in cubic III-V binary compounds and comparable to pressure coefficients for direct band gap in hexagonal III-N materials^43^, see Fig. 6. In addition, it is visible that the pressure coefficients are different for A and B transitions. Moreover it is worth noting that the pressure coefficient for indirect gap (X transition) is negative (see Fig. 5). It means that a semiconductor to metal transition is expected for MoS_2_, MoSe_2_, WS_2_, and WSe_2_ crystals at some pressures. These pressures have been estimated to be 140, 180, 190, and 240 kbar for MoS_2_, MoSe_2_, WS_2_, and WSe_2_, respectively. These values are not directly measured and are much larger than the maximal hydrostatic pressures we were able to apply, which in our case results from technical limitations. We are confident that further experimental studies are necessary to verify these predictions and study the semiconductor to metal transition in these materials, however, our predictions derived from the pressure coefficients may serve as a solid starting point for future, more detailed research of this property.

**Figure 7:**
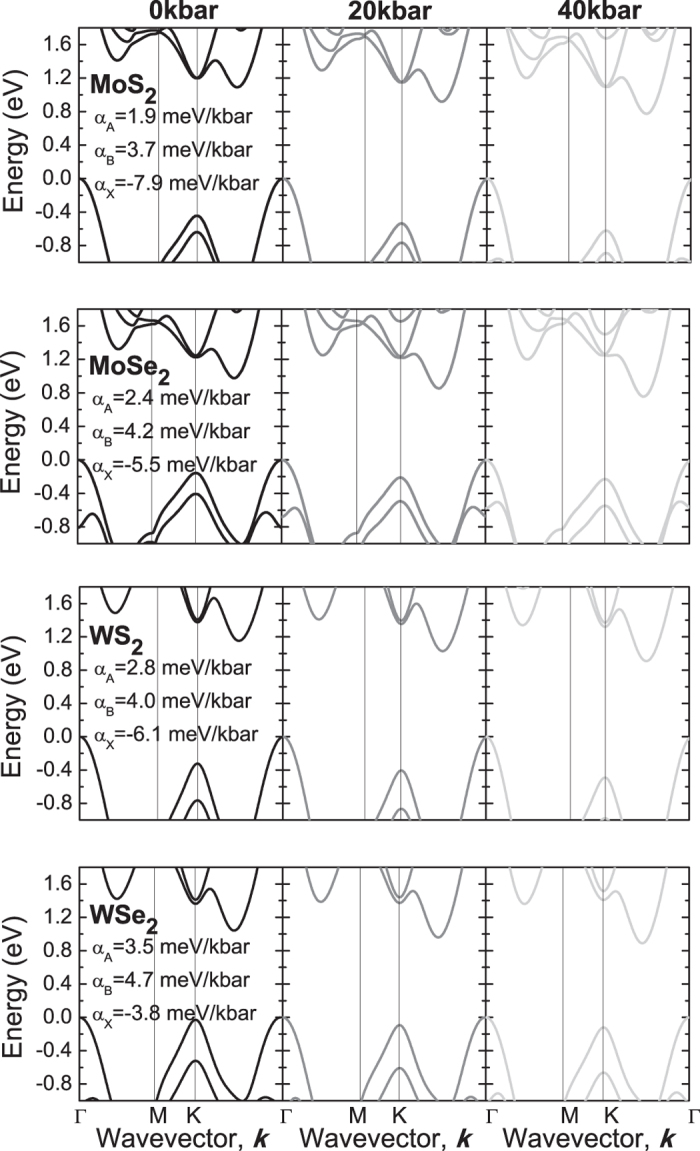
Electronic band structures for MoS_2_ (**a**), MoSe_2_ (**b**), WS_2_ (**c**), and WSe_2_ (**d**) crystals obtained for various hydrostatic pressures: 0, 20, and 40 kbar, calculated with MBJLDA functional and GGA + DFT-D3 geometry optimization.

The pressure induced evolution of the electronic band structure for the four crystals is plotted in Fig. 7. It is clearly visible that the hydrostatic pressure influences the electronic band structure of MoS_2_, MoSe_2_, WS_2_, and WSe_2_ crystals very strongly, narrowing the band gap with the increase in hydrostatic pressure, and at a hydrostatic pressure high enough, can close the band gap completely creating a metallic material. As seen in Fig. 7 the narrowing of band gap for the four crystals is due to a down shift of the conduction band between K and Γ point and an up shift of the valence band at the Γ point relatively to the valence band maximum at the K point. Changes at these places of Brillouin zone are also responsible for the change in the character of band gap from indirect to direct with the reduction of number of monolayer from bulk regime to a single layer^9^. It suggests that the inter-layer interaction in these crystals is the factor which strongly influences the electronic band structure at these points. The external hydrostatic pressure influences both the distance between atoms in the layer and the distance between layers leading to the electronic band structure with very narrow indirect gap for high hydrostatic pressures. For regular III-V semiconductors the semiconductor to metal transition has not been observed since the hydrostatic pressure opens the band gap, which for most III-V semiconductors is defined at the Γ point of Brillouin zone. The character of the band gap can change from direct to indirect with the increase in hydrostatic pressure due to the down shift of the conduction band outside of the Γ point of Brillouin zone (for example in GaAs it is X point) but hydrostatic pressures needed to close the energy gap in III-V semiconductors would be very high if at all be possible. Therefore a semiconductor to metal transition in regular III-V semiconductors is not expected in the range of hydrostatic pressure ~200 kbar. In TMDs studied in this paper the band gap is indirect and is defined outside the Γ point of Brillouin zone due to lower symmetry of TMDs crystals in comparison to cubic III-V compounds. Moreover this energy gap is narrower than the energy gap between the valence band maximum and the conduction band at X point in III-V semiconductors. Therefore it is possible to achieve a semiconductor to metal transition in these materials by applying hydrostatic pressure.

DFT calculations of this family of materials are especially demanding. It is a common knowledge that there are certain tasks where standard DFT (within the LDA or GGA functionals) fail. These tasks include a proper description of the band gap and van der Waals interactions. The systems we study here are an open band gap semiconductors with layers held together by van der Waals interactions. Therefore a proper computational approach has to be taken in order to describe their properties correctly. Hence, the van der Waals interactions in our main calculations have been included by the means of the DFT-D3 dispersion correction proposed by Grimme *et al*.^44^ and for the correction of the band gap, the MBJLDA exchange correlation functional was used. However, in this paper, to provide more meaningful insight into the matter of calculating the pressure dependence of the band structure, the electronic band structures for MoS_2_, MoSe_2_, WS_2_, and WSe_2_ crystals and their pressure dependence have been calculated with other approaches as well and show how both of these corrections impact and improve the overall results. In all band structure calculations the spin-orbit interaction was included, since it becomes especially important in the materials with heavier elements such as tungsten.

For the geometry optimization of the structure we first used the standard LDA^45^ and GGA (PBE)^46^ functionals but we found that not only the lattice parameters were incorrect but also the layer’s thickness was wrong. The LDA functional gave a reasonable c/*a* ratio but the values of the lattice constants and the layer thickness were slightly underestimated. GGA on the other side not only overestimated the parameters but also gave a very wrong c/*a* ratio (c lattice parameter highly overestimated). This was expected since it is widely known that these functionals do not describe the van der Waals interactions correctly. Thus we employed the DFT-D3 dispersion correction as proposed by Grimme *et al*.^44^. Together with the GGA functional, this correction performed excellently and allowed us to obtain a perfect match of the lattice parameters, c/*a* ratio and layer thickness with the available experimental data. A summary of all results for different approaches to the geometry optimization (at equilibrium pressure only) are presented in Table 1 together with experimental data^47^,^48^,^49^,^50^. For obtaining the band structure we continued the calculations with pure GGA and LDA functionals, despite their failure in correct geometry optimization, aware at the same time that they will also fail in describing the band gap. This was done in order to provide the results of these most commonly used functionals, show their inevitable failure in these kinds of systems, and alert the readers about their shortcomings. As expected, the values of the band gaps were underestimated. The GGA functional performed especially badly by not only lowering the band gap but also distorting the band widths and energy separation between the indirect and direct band gap. The LDA performed slightly better, keeping the band widths more reasonable but the band gap still severely underestimated. The failure of the GGA functional is understandable, since the geometry optimization in this case is very wrong. The use of incorrect structural parameters causes distortions of the band gap much like those induced by strain, which strongly affects the band shapes. A proper geometry optimization is crucial in obtaining a correct band structure. A pure GGA functional was also used to determine the band structures with the correct geometries obtained with GGA and DFT-D3 which, as expected, improved the results in terms of the shape of the bands but did not improve the band gaps themselves. After that, in order to more correctly describe the band structure we used the MBJLDA functional which is known for a significant improvement over standard LDA and GGA while keeping the computational time reasonable^51^,^52^. This was done for all the geometries obtained earlier. The MBJLDA functional indeed improved the overall band structures, especially the lowest, indirect band gap and the energy separation between the direct and indirect gap (the band shape/width). In each case, however, the band gap remained slightly underestimated, which is visible in Fig. 5. This could be easily improved by increasing the “c” parameter in the functional^53^. However, we intentionally kept the self consistently calculated “c” parameter unchanged in order to stay as independent of the experimental results as possible for the comparison of the theoretical and experimental approaches to be more meaningful. We chose the relative change in the gaps and the change rate of their values as the most important quantities, and in this case the slight underestimation of the band gap value is unimportant, keeping in mind that in the MBJLDA approach the shape of the bands improved significantly. It is worth noticing, that the difference between the band structures obtained within the MBJLDA functional with LDA geometry and GGA + DFT-D3 geometry are very similar. This means that the slight underestimation of the lattice parameters within LDA does not affect the band structure that much and it is the c/*a* ratio that is much more important. A summary of all results for different approaches to the band structure calculations with different methods of geometry optimization (at equilibrium pressure only) are presented in Table 2 with the most reliable results in bold.

Table 3 presents the pressure dependence of the band structure for different approaches and experimental results of our PR spectroscopy. As we expected and hoped for, the most advanced and technically correct approach for the type of systems with an open band gap and significant role of the van der Waals interaction, an approach where the GGA geometry was corrected for the van der Waals interaction by the DFT-D3 method and the band structure was calculated with the MBJLDA functional, yielded the best results of the pressure dependence of the band structure. The agreement of the results of this approach with the experimental data is in fact remarkable. Much to our surprise, the LDA geometry optimization with MBJLDA band structure, our method of choice when calculating the band structure of regular III-V, II-VI and IV group semiconductors^54^,^55^, performed excellently in the description of the pressure dependence as well, despite the improper description of the van der Waals interactions in geometry optimization. The other approaches, as expected, failed miserably in the description of this type of computationally demanding system. The best approach, namely the GGA + DFT-D3 geometry optimization combined with the MBJLDA band structure is highlighted in bold in this table.

The electronic band structures plotted in Figs 2 and 7 have been obtained with the most successful approach described above. Table 3 summarizes experimental data and theoretical predictions obtained within various approaches with the column with the method of choice in bold. Using the results of the most accurate method and taking into account the uncertainty in determination of the calculated band gap, we have estimated that the semiconductor to metal transition for MoS_2_, MoSe_2_, WS_2_, and WSe_2_ crystals is expected at 140, 180, 190, and 240 kbar, respectively. These pressures are very consistent with recent studies of semiconductor to metal transition in MoS_2_ and WS_2_, although slightly underestimated. This was expected and can be easily explained: the DFT calculated values of the band gap itself are slightly too small, and the character of the changes is probably not exactly linear in higher pressures which are not studied in this paper. Further optical studies of both bulk and monolayer TMDs at high hydrostatic pressures (>20 kbar) by PR and absorption should allow more quantitative analysis and lead to additional insight into these unique changes in the electronic band structure of TMDs.

## Conclusions

The present work shows experimental and theoretical studies of pressure induced changes in the electronic band structure of MoS_2_, MoSe_2_, WS_2_, and WSe_2_ crystals. Pressure coefficients have been determined experimentally for A and B transitions in MoS_2_, MoSe_2_, WS_2_, and WSe_2_ crystals in the pressure range <20 kbar. Values of these coefficients have been found to be in very good agreement with theoretical predictions obtained within DFT calculations. The pressure coefficients for indirect gap have been found to be negative and it has been estimated that the semiconductor to metal transition for MoS_2_, MoSe_2_, WS_2_, and WSe_2_ crystals is expected at around 140, 180, 190, and 240 kbar, respectively. In addition, a comparative study of different computational DFT approaches has been performed and analyzed.

## Methods

### Sample growth

MoS_2_, MoSe_2_, WS_2_, and WSe_2_ samples were grown by iodine assisted vapor transport technique at high temperatures (900–1100 °C) and low pressures (~1E-6 Torr) in a sealed quartz ampoules. During growth ~50 °C temperature differential created between hot and cold zones to initiate nucleation and facilitate precursor transport. Prior to growth quartz ampoules (~15 cm in length, 2.4 cm outer diameter, 2.0 inner diameter) were cleaned in piranha solution and annealed in H_2_ gas to remove contaminants. Precursors (Mo, W foils and S, Se nuggets) were mixed in 1:2.05 M:X stoichiometric ratio and iodine pieces were added as a transport agent. Quartz ampoule is sealed under vacuum (1 microTorr). Synthesized crystals were characterized by Raman/PL spectroscopy and XRD measurements.

### Photoreflectance measurements at high hydrostatic pressures

For PR measurements at high hydrostatic pressures the samples were mounted on the plug with a sapphire window inside a pressure cell^56^. The pressure was increased by pushing the piston inwards the cell body. The liquid used as a pressure medium was Daphne 7474 remained hydrostatic and transparent in the whole pressure range (20 kbar). The pressure was determined by changes of the resistance of an InSb sensor which gives about 0.1 kbar sensitivity in the 20 kbar range. A single grating of 0.55 meter focal-length monochromator and a Si *pin* photodiode were used to disperse and detect the light reflected from the samples. A 150W tungsten-halogen bulb was used as the probe, and a semiconductor laser (405 nm line) was used as the pump source. The pump beam was modulated by a mechanical chopper at a frequency of 280 Hz. Phase-sensitive detection of the PR signal was performed using a lock-in amplifier. Other relevant details on PR measurements at high hydrostatic pressure can be found in ref. 56.

### Theoretical calculations

The DFT calculations have been performed with a modified version of the ABINIT code^57^. The geometry optimization has been performed with the use of PAW atomic datasets generated with the ATOMPAW package^58^. This representation of the atoms is believed to yield the most correct results, since it includes the core electrons as well. In order to obtain the optimized shape (a/c ratio), size and atomic positions corresponding to a certain hydrostatic pressure we used the stress tensor as a target for geometry optimization. A diagonal stress tensor with equal values on the diagonal was used, as it corresponds to the case of externally applied hydrostatic pressure. Then, the optimized geometries were used for regular band structure calculations. A proper geometry optimization is crucial in determining other properties correctly, so especially strict convergence criteria (forces [stresses] could not exceed 10^−11^ Hartree/Bohr [10^−8^ Hartree/Bohr^3^]) have been used. The band structures have been calculated with the use of fully relativistic pseudopotentials, with the spin-orbit interactions included, generated with the APE package^59^ especially for this study. Different pseudopotentials have been generated for each functional tested here, with all other parameters (e.g. the cut-off radii) left unchanged. This is the proper approach, since using a pseudopotential generated with a different functional than the one used in calculations can create misleading and wrong results. A total energy difference of 10^−10^ eV and a wavefunction squared residual of 10^−13^ have been used as a convergence criteria in the band structure calculations. In all calculations a 10 × 10 × 6 Monkhorst-Pack mesh was used^60^, as a result of convergence studies. We have checked that a denser mesh does not change our results.

## Additional Information

**How to cite this article**: Dybała, F. *et al*. Pressure coefficients for direct optical transitions in MoS_2_, MoSe_2_, WS_2_, and WSe_2_ crystals and semiconductor to metal transitions. *Sci. Rep.* **6**, 26663; doi: 10.1038/srep26663 (2016).

## Figures and Tables

**Table 1:** 

Material	GGA + DFT-D3	GGA	LDA	Experiment
MoS_2_				
c (Å)	**12.28**	14.99	12.11	12.32^a^, 12.30^b^
c/*a*	**3.89**	4.71	3.87	3.89^a^, 3.89^b^
MoSe_2_				
c (Å)	**12.88**	15.32	12.71	12.93^c^
c/*a*	**3.92**	4.61	12.19	3.93^c^
WS_2_				
c (Å)	**12.32**	14.59	12.19	12.32^d^
c/*a*	**3.89**	4.57	3.89	3.91^d^
WSe_2_				
c (Å)	**12.95**	15.05	12.79	12.96^d^
c/*a*	**3.94**	4.53	3.93	3.95^d^

Lattice parameters (*a* lattice constant and c/*a* ratio) obtained within different DFT functionals (equilibrium pressure) compared with experimental literature data. The most accurate method is highlighted in bold.

^a^Ref. 46.

^b^Ref. 47.

^c^Ref. 48.

^d^Ref. 49.

**Table 2:** 

Method	MoS_2_ A transition (eV)	MoS_2_ B transition (eV)	MoS_2_ X transition (eV)
PR	1.855	2.065	NA
MBJLDA			
(GGA + DFT-D3)	1.64	1.84	1.09
(LDA)	1.73	1.94	1.04
GGA			
(GGA + DFT-D3)	1.65	1.88	0.85
(GGA)	1.61	1.77	1.39
LDA (LDA)	1.73	1.99	0.72
	****MoSe**_**2**_ A transition (meV/kbar)**	**MoSe**_**2**_ **B transition (meV/kbar)**	****MoSe**_**2**_ X transition (meV/kbar)**
PR	1.555	1.812	NA
MBJLDA			
(GGA + DFT-D3)	1.39	1.63	0.98
(LDA)	1.45	1.72	0.94
GGA			
(GGA + DFT-D3)	1.39	1.68	0.79
(LDA)	1.34	1.53	1.34
LDA	1.46	1.77	0.70
	****WS**_**2**_ A transition (meV/kbar)**	**WS**_**2**_ **B transition (meV/kbar)**	****WS**_**2**_ X transition (meV/kbar)**
PR	1.993	2.432	NA
MBJLDA			
(GGA + DFT-D3)	1.70	2.14	1.15
(LDA)	1.79	2.23	1.12
GGA			
(GGA + DFT-D3)	1.63	2.09	0.92
(GGA)	1.54	1.98	1.49
LDA (LDA)	1.68	2.16	0.81
	****WSe**_**2**_ A transition (meV/kbar)**	**WSe**_**2**_ **B transition (meV/kbar)**	****WSe**_**2**_ X transition (meV/kbar)**
PR	1.647	2.095	NA
MBJLDA			
(GGA + DFT-D3)	1.39	1.88	1.04
(LDA)	1.47	1.97	1.01
GGA			
(GGA + DFT-D3)	1.33	1.85	0.86
(GGA)	1.25	1.72	1.17
LDA (LDA)	1.38	1.92	0.77

Energies of the three studied optical transitions calculated theoretically with different DFT functionals (equilibrium pressure) together with energies of A and B transitions extracted from PR measurements. Different combinations of functionals are presented here so in the method column, the first one, without parentheses, is the functional used for band structure calculations and those in parentheses are functionals used with it for geometry optimization.

**Table 3:** 

Method	MoS_2_ α_A_ for A transition (meV/kbar)	MoS_2_ α_B_ for B transition (meV/kbar)	MoS_2_ α_X_ for X transition (meV/kbar)
PR	**2.0**	**3.6**	NA
**MBJLDA**			
**(GGA** + **DFT-D3)**	**1.94**	**3.73**	**−7.90**
(LDA)	2.00	3.69	−7.27
GGA			
(GGA + DFT-D3)	2.02	3.57	−2.78
(GGA)	1.91	3.91	−12.4
LDA (LDA)	1.74	3.73	−5.61
	****MoSe**_**2**_ A transition (meV/kbar)**	**MoSe**_**2**_ **B transition (meV/kbar)**	****MoSe**_**2**_ X transition (meV/kbar)**
PR	**2.3**	**4.1**	NA
**MBJLDA**			
**(GGA** + **DFT-D3)**	**2.44**	**4.15**	**−5.51**
(LDA)	2.04	3.95	−7.03
GGA			
(GGA + DFT-D3)	2.08	3.60	−4.62
(GGA)	1.97	4.30	−13.8
LDA (LDA)	0.70	2.74	4.68
	****WS**_**2**_ A transition (meV/kbar)**	**WS**_**2**_ **B transition (meV/kbar)**	****WS**_**2**_ X transition (meV/kbar)**
PR	**2.7**	**4.1**	NA
**MBJLDA**			
**(GGA** + **DFT-D3)**	**2.83**	**3.95**	**−6.11**
(LDA)	2.78	4.42	−7.48
GGA			
(GGA + DFT-D3)	2.89	3.94	−2.62
(GGA)	2.68	3.81	−12.9
LDA (LDA)	1.69	3.40	−5.11
	****WSe**_**2**_ A transition(meV/kbar)**	**WSe**_**2**_ **B transition (meV/kbar)**	****WSe**_**2**_ X transition (meV/kbar)**
PR	**3.5**	**5.3**	NA
**MBJLDA**			
**(GGA** + **DFT-D3)**	**3.45**	**4.71**	**−3.79**
(LDA)	3.31	5.37	−3.25
GGA			
(GGA + DFT-D3)	3.11	4.14	−3.32
(GGA)	2.78	3.95	−7.59
LDA (LDA)	0.61	2.25	−4.25

Pressure coefficients of all studied TMD materials obtained experimentally, as well as within all the studied DFT approaches. The method of choice where the deviation from the experimental was the smallest is highlighted in bold. Different combinations of functionals are presented here so in the method column, the first one, without parentheses, is the functional used for band structure calculations and those in parentheses are functionals used with it for geometry optimization.
